# Characterization of metastatic tumor antigen 1 and its interaction with hepatitis B virus X protein in NF-κB signaling and tumor progression in a woodchuck hepatocellular carcinoma model

**DOI:** 10.18632/oncotarget.9986

**Published:** 2016-06-13

**Authors:** Yung-Tsung Li, Chun-Jen Liu, Tung-Hung Su, Huei-Ru Cheng, Yung-Ming Jeng, Hsiu-Lin Lin, Chih-Chiang Wang, Jia-Horng Kao, Pei-Jer Chen, Ding-Shinn Chen, Hui-Lin Wu

**Affiliations:** ^1^ Graduate Institute of Clinical Medicine, College of Medicine, National Taiwan University, Taipei, Taiwan; ^2^ Department of Internal Medicine, National Taiwan University Hospital, Taipei, Taiwan; ^3^ Hepatitis Research Center, National Taiwan University Hospital, Taipei, Taiwan; ^4^ Graduate Institute of Pathology, College of Medicine, National Taiwan University, Taipei, Taiwan; ^5^ Department of Pathology, National Taiwan University Hospital, Taipei, Taiwan

**Keywords:** metastatic tumor antigen 1 (MTA1), hepatitis B virus (HBV), hepatocellular carcinoma (HCC), woodchuck, splicing variant

## Abstract

The metastatic tumor antigen 1 (MTA1) protein is associated with tumor invasiveness and poor prognosis in patients with hepatocellular carcinoma (HCC), particularly in those with hepatitis B virus (HBV)-related HCC. Chronically woodchuck hepatitis virus (WHV)-infected woodchuck is an ideal animal model for studying the pathogenesis of HBV-associated liver diseases, including HCC. To investigate the roles of MTA1 in HBV-associated hepatocarcinogenesis in the woodchuck model, we cloned the woodchuck MTA1 (wk-MTA1) complementary (c)DNA and characterized its molecular functions. The sequence and organization of the wk-MTA1 protein were highly conserved among different species. Similar to its expression in human HCC, wk-MTA1 was upregulated in woodchuck HCC, as determined at RNA and protein levels. Furthermore, an MTA1-spliced variant, wk-MTA1dE4, was overexpressed in woodchuck HCC, and it was attributed to approximately 50% of the total transcripts. The percentage of wk-MTA1dE4-overexpressed woodchuck HCCs was higher than that of the total wk-MTA1-overexpressed HCCs (77.8% vs 61.1%) and wk-MTA1dE4 may represent a more sensitive marker than the total wk-MTA1 in woodchuck HCC. We overexpressed or knocked down wk-MTA1 in a woodchuck HCC cell line and demonstrated that wk-MTA1 could interact with the WHV X protein (WHx) and play indispensable roles in WHx-mediated NF-κB activation and tumor cell migration- and invasion-promoting activities. In conclusion, our results support the hypothesis that woodchuck HCC recapitulates HBV-associated HCC with respect to the molecular characteristics of MTA1 and provides new clues for conducting mechanistic studies of MTA1 in HBV-associated hepatocarcinogenesis, including the possible clinical significance of wk-MTA1dE4.

## INTRODUCTION

Hepatocellular carcinoma (HCC) is one of the most lethal cancers worldwide, with an estimated 0.5–1 million new cases annually [1, 2]. The lack of sensitive biomarkers for timely diagnosis and effective therapeutics for advanced tumors are the 2 major reasons for the poor outcome of HCC [3–5]. The molecular mechanism underlying HCC is fairly heterogeneous among patients, and several mechanisms of tumorigenesis seem to be involved, including chromatin remodeling, cell differentiation, cell proliferation, growth factor signaling, and angiogenesis [5, 6]. Therefore, unraveling the molecular mechanisms of HCC is crucial for early diagnosis, prognosis prediction, and the development of personalized therapeutic strategies.

Metastatic tumor antigen 1 (MTA1) protein is an integral component of the nucleosome remodeling histone deacetylase complex involved in regulating transcription and chromatin remodeling [7]. The overexpression of the *MTA1* gene family has been reported in numerous cancers, including breast, esophageal, gastric, pancreatic, and colorectal cancers and HCC [8–10]. Previous studies have reported that MTA1 overexpression is closely correlated with microvascular invasion, frequent postoperative recurrence, and poor prognosis as well as linked to the degree of intrahepatic invasion and metastasis in patients with HCC [11, 12]. In addition, the cumulative survival rate of patients with MTA1-positive HCC was significantly lower than that of patients without MTA1 [11]. These clinical observations indicated that MTA1 is strongly associated with hepatocarcinogenesis and malignant features.

Both chronic hepatitis B virus (HBV) and hepatitis C virus (HCV) infections are major risk factors for HCC [13, 14]. The frequency of MTA1 overexpression is much higher in HBV-associated HCC (HBV–HCC) than in HCV-associated HCC [11]; however, the mechanisms underlying this observation remain elusive. The direct effects of HBV and indirect effects of inflammation, regeneration, and cirrhosis contribute to the development of HBV–HCC [15, 16]. Few studies have reported the potential role of MTA1 in HBV-associated hepatocarcinogenesis [17–19]. MTA1 has been demonstrated to play an integral role in the Hepatitis B virus X (HBx) protein stimulation of nuclear factor-kappa B (NF-κB) signaling, stabilization of hypoxia-inducible factor-1 alpha, and induction of inducible nitric oxide expression. HBx is a crucial oncoprotein of HBV and is involved in numerous mechanisms of oncogenesis [20]; therefore, the interaction between MTA1 and HBx might significantly contribute to HCC development.

To further explore the clinicopathological significance and potential clinical applications of MTA1, appropriate animal models are required. To date, animal models for HCC have mainly been established in small rodents. Because HBV has an extremely limited host range and cannot infect small rodents, such as mice and rats, chemically induced or implanted HCCs in these conventional animals cannot adequately represent HBV–HCC, in which tumors develop because of a chronic HBV infection. Among the animal models for HBV, woodchucks that are chronically infected with woodchuck hepatitis virus (WHV) is recognized as a clinically relevant animal model for studying HBV-associated diseases, including HCC. Therefore, in this study, we examined the appropriateness of using the woodchuck model for studying the roles of MTA1 in HBV–HCC with respect to MTA1 overexpression and molecular interaction with the hepadnavirus X protein. Furthermore, we explored the significance of MTA1 expression in the malignant characteristics of HCC, including migration and invasiveness.

## RESULTS

### Cloning and sequence analysis of woodchuck MTA1 cDNA

**Figure 1:**
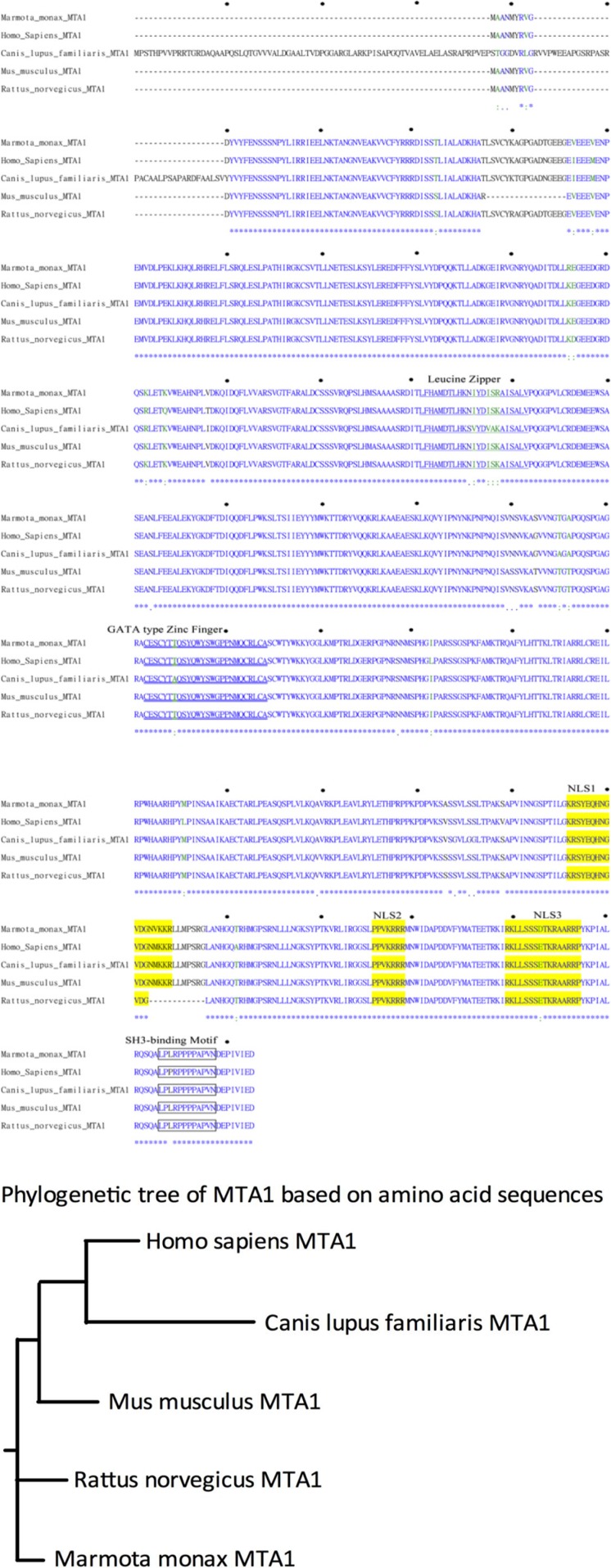
Comparison of the deduced MTA1 amino acid sequences of different species (**A**) Alignment of the deduced wk-MTA1 amino acid sequence with the sequences of other species, including humans (NM_004689.3), dogs (XM_537568.2), mice (NM_054081.2), and rats (NM_022588.1). Asterisks (*) indicate conserved amino acid identity; colons (:) and periods (.) indicate conservative and semiconservative substitutions, respectively, and hyphens (−) indicate the absence of amino acids. The amino acid sequences corresponding to the leucine zipper, GATA-type zinc finger, nuclear localization signal, and SH3-binding motif sequences are underlined, double underlined, shaded in yellow, and boxed, respectively. Moreover, the amino acid sequence of wk-MTA1 was submitted to GenBank (JX891465.1). (**B**) Phylogenetic analysis of the MTA1 amino acid sequences of different mammalian species. This rooted phylogenetic tree was constructed using the ClustalW1.81 program.

To examine the roles of wk-MTA1 in hepadnavirus-associated hepatocarcinogenesis in the woodchuck model, we first cloned the full-length wk-MTA1 cDNA by using polymerase chain reaction (PCR)-based strategies with primers designed according to the highly conserved sequences among several mammalian MTA1 cDNAs. Three DNA fragments with overlapping sequences, namely nt −15 to +1244; nt +1072 to +1718; and nt +1320 to 3′ untranslated region (UTR), were obtained using slowdown PCR, 5′ RLM-RACE and 3′ RLM-RACE, respectively (Figure S1A). The sequence of 2644 nucleotides covering a short portion of the 5′ UTR, the entire coding region, and 3′ UTR for wk-MTA1 cDNA was assembled (Figure S1B) and submitted to GenBank (JX891465.1). In addition to the full-length wk-MTA1 cDNA, we obtained 2 naturally occurring alternative splice variants, wk-MTA1dE4 and wk-MTA1dE18 (Figure S1D), during the cloning process. A database homology search and phylogenetic tree analysis revealed that wk-MTA1 was highly homologous to the MTA1 of other species at both nucleotide and amino acid levels (Figures 1B and S1C and Table 1), with 92% and 97% homology to the nucleotide and amino acid levels of human MTA1, respectively.

**Table 1:** Comparison of the wk-MTA1 sequence with the respective sequences of other species regarding the nucleotide and amino acid levels

Species	Homology (%)	
	Nucleotide	Amino acid
Human	92	97
Monkey	90	96
Horse	90	95
Dog	89	95
Rat	90	98
Mouse	90	98
Chicken	78	87
Fish	71	80
Fly	27	49

The full-length wk-MTA1 cDNA encodes a protein of 715 amino acids, which contains several conserved functional motifs, including leucine zipper motif, putative zinc finger DNA binding motif, possible *src* homology 3 domain-binding site (*SH3*-binding), 3 putative nuclear localization signals, and proline-rich stretch (LPPRPPPPAP) at its carboxy-terminal end. The conservation of these crucial structural motifs in wk-MTA1 indicates that wk-MTA1 shares structural and functional similarities with human MTA1.

### Wk-MTA1 and its splice variants are overexpressed in WHV-infected woodchuck HCC tissues

**Figure 2:**
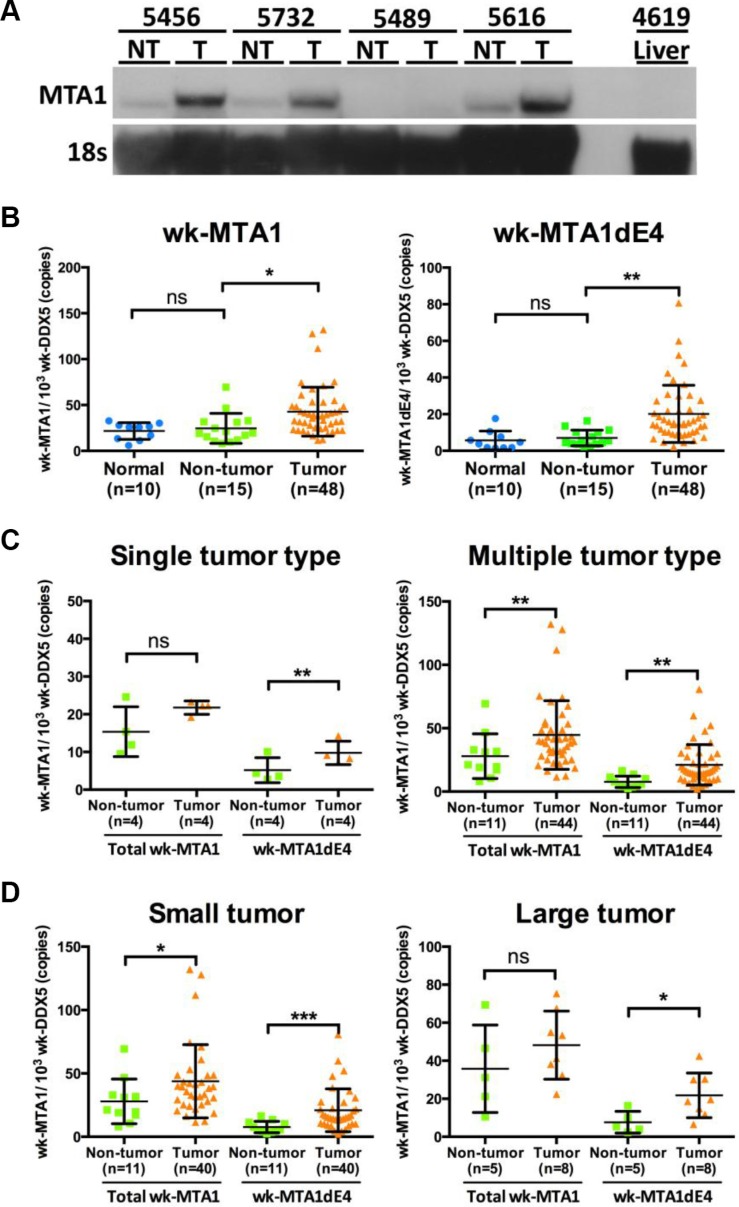
Expression of wk-MTA1 mRNA in normal, nontumor, and tumor liver tissues from woodchuck autopsy samples (**A**) Northern blotting analysis of woodchuck MTA1 RNA in liver tissues. MTA1 mRNA was detected using a DIG-labeled MTA1 probe covering the full-length wk-MTA1 from nt +392 to +910 (the start codon is designated as +1). Liver tissues were obtained from one WHV-resolved woodchuck (designated as 4619 liver) and from 4 woodchucks with paired tumor and nontumor tissues (designated as T and NT, respectively). Moreover, 18S RNA was detected using a DIG-labeled hybridization probe and served as the loading control. (**B**) The expression levels of the total wk-MTA1 and wk-MTA1dE4 were quantitated using qRT-PCR in normal, nontumor, and tumor liver tissues. Normal liver tissues were obtained from 10 WHV-resolved woodchucks. All tumor and corresponding nontumor tissues were obtained from woodchucks that were chronically infected with WHV. The results are presented as mean ± SD. The association was assessed by Student's *t*-test (*P < .05; **P < .01). (**C, D**) The expression levels of the total wk-MTA1 and wk-MTA1dE4 in tumors of different characteristics and in their corresponding nontumor liver tissues were determined using qRT-PCR. Tumor volume over 65 cm^3^ (the estimated tumor volume of a tumor with a 5-cm diameter) was arbitrarily designated as large. The tumor volume was calculated using the following formula: volume = shortest axis^2^ × longest axis × 0.52 [35]. The expression level of each target gene was normalized to that of wk-DDX5. The results are presented as mean ± SD. The association was assessed by the one-way repeated measured ANOVA (ns: not significant, *P < .05; **P < .01, ***P < .001).

We first used Northern blotting and quantitative reverse transcription-PCR (qRT-PCR) for determining the expression level of the wk-MTA1 transcript in woodchuck liver tissues. Northern blotting revealed a single band of approximately 3.0 kb (Figure 2A). Similar to human MTA1 RNA, wk-MTA1 RNA was scarcely detected in the normal liver tissues, and its expression level was much higher in tumor than in nontumor cells in most tissues. Consistent with the results of Northern blotting, the expression level of wk-MTA1 was significantly higher in tumor tissues than in the adjacent nontumor tissues or normal liver tissues, as assessed using qRT-PCR [mean (± standard deviation; SD): 42.69 (± 26.67) vs 24.58 (± 16.29) and 21.69 (± 9.09) wk-MTA1/10^3^-wk-DDX5 copies, respectively; *P* < .05] (Figure 2B).

Furthermore, we used conventional RT-PCR with primers specific to wk-MTA1dE4 and wk-MTA1dE18 for detecting these spliced variants in woodchuck liver tissues. We observed a notably high expression level of wk-MTA1dE4, particularly in tumor tissues, accounting for approximately 50% of the total wk-MTA1 (Figure S2). In contrast, the level of wk-MTA1dE18 is quite low in woodchuck liver tissues. Therefore, we focused our study on wk-MTA1dE4 and designed wk-MTA1dE4-specific qRT-PCR to quantitate its expression in more woodchuck liver tissues. The results revealed wk-MTA1dE4 overexpression in tumor tissues compared with the expression in nontumor and normal liver tissues [mean (± SD): 20.16 (± 15.59) vs 7.01 (± 4.3) and 5.68 (± 5.14) wk-MTA1dE4/10^3^ wk-DDX5 copies, respectively; *P* < .01; Figure 2B]. To explore the potential clinical significance of wk-MTA1dE4, we assessed the percentage of wk-MTA1dE4 overexpression in woodchuck HCCs and further correlated the expression levels of the total wk-MTA1 and wk-MTA1dE4 with different tumor characteristics. We noted the percentage of wk-MTA1dE4-overexpressed woodchuck HCCs was higher than that of the total wk-MTA1-overexpressed HCCs (77.8% vs 61.1%). Moreover, overexpression of wk-MTA1dE4 was associated with all tumor types. In contrast, total wk-MTA1 was only found to be associated with multiple type and a smaller tumors (Figure 2C and 2D).

**Figure 3:**
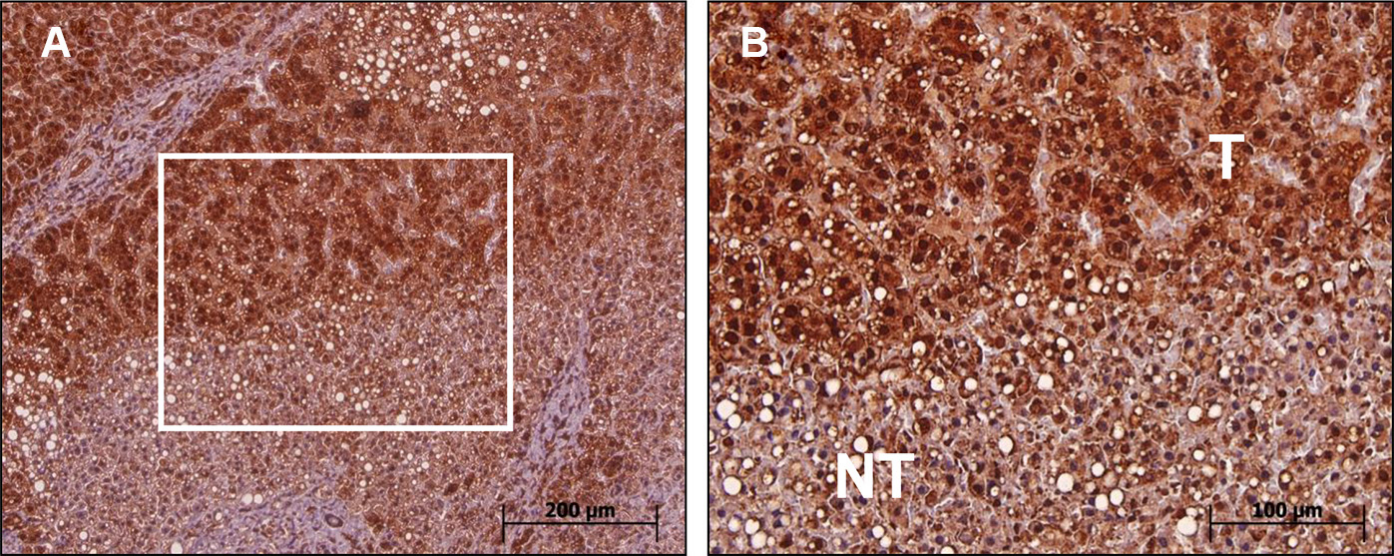
Immunohistochemical analysis of wk-MTA1 expression in tumor and nontumor cells of liver tissues (**A**) Most tumor cells exhibit a strong wk-MTA1 staining compared with nontumor cells in the same tissue section. Original magnification: ×100. (**B**) Enlarged picture of the boxed region in (A). Original magnification: ×200.

We used immunohistochemical (IHC) staining for studying MTA1 expression in woodchuck HCC with a commercially available antihuman MTA1 antibody crossreacting to wk-MTA1. Consistent with the RNA analysis results, the MTA1 protein was also overexpressed in woodchuck HCC. Liver tumor cells exhibited an intense positive staining for wk-MTA1 in both nuclear and cytoplasmic compartments, whereas nontumor liver cells only exhibited weak staining for wk-MTA1 (Figure 3B). In tumor cells, wk-MTA1 staining was much denser in the nucleus than in the cytoplasm. Furthermore, most tumor cells exhibited a higher nucleus:cytoplasm ratio and hyperchromatic nuclei than did nontumor cells.

### Wk-MTA1 is induced by WHx and plays an essential role in WHx-mediated wk-NF-κB activation

Similar to human MTA1 expression in HBV–HCC, our data also indicated wk-MTA1 overexpression in woodchuck WHV-related HCC. A previous study has demonstrated that oncogenic viral protein, HBx, could upregulate MTA1 expression in human HCC cells (HepG2) and that MTA1 plays a critical role in HBx-mediated NF-κB activation [19], which may contribute to the carcinogenesis of HCC. This observation prompted us to investigate whether wk-MTA1 could also recapitulate the molecular activity of human MTA1 with respect to its interaction with the viral X protein. The results revealed that woodchuck hepatitis virus X (WHx) could dose-dependently upregulate wk-MTA1 expression in the woodchuck hepatoma cell line WCH17 (Figure S3).

**Figure 4:**
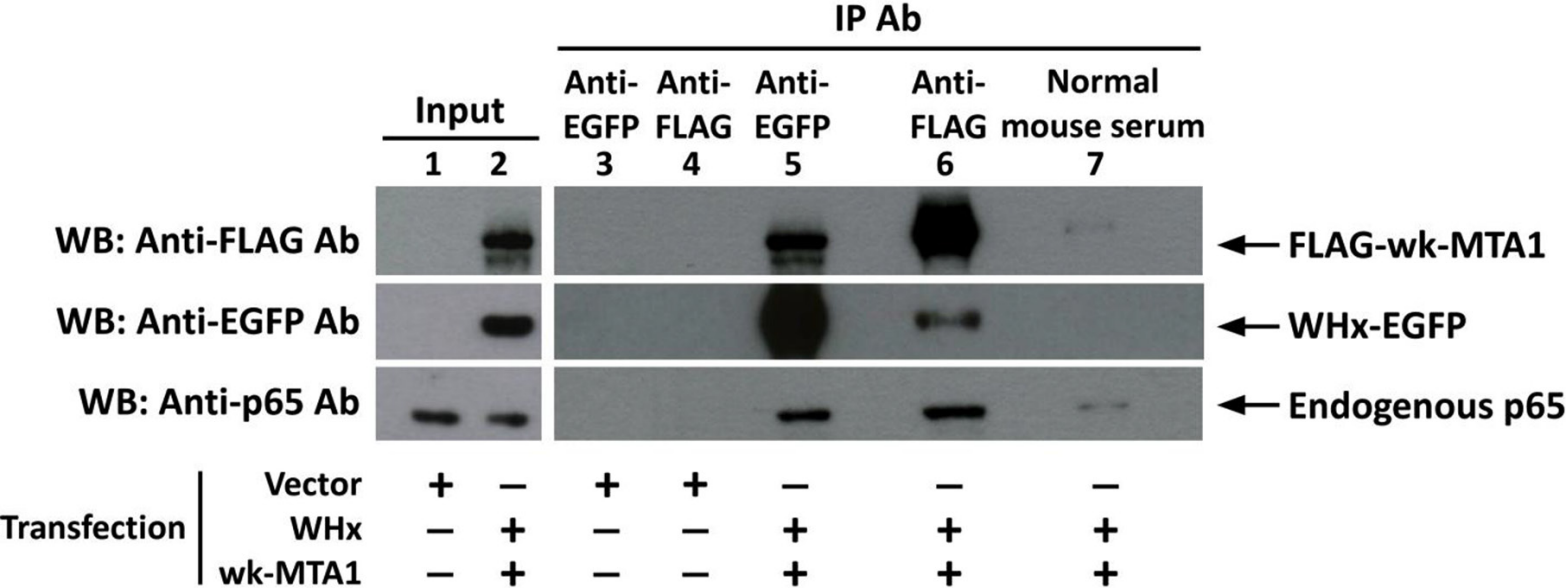
Wk-MTA1 could interact with the WHx and p65 of NF-κB in 293T cells We transfected 293T cells with WHx-EGFP and flag-wk-MTA1 expressing plasmids (lanes 2 and 5–7) or with empty vectors (lanes 1, 3, and 4). Whole-cell lysates were subjected to immunoprecipitation with monoclonal anti-GFP, anti-FLAG antibody, or normal mouse serum. The presence of target proteins in input lysates or immunoprecipitates was detected using immunoblotting with specific antibodies.

**Figure 5:**
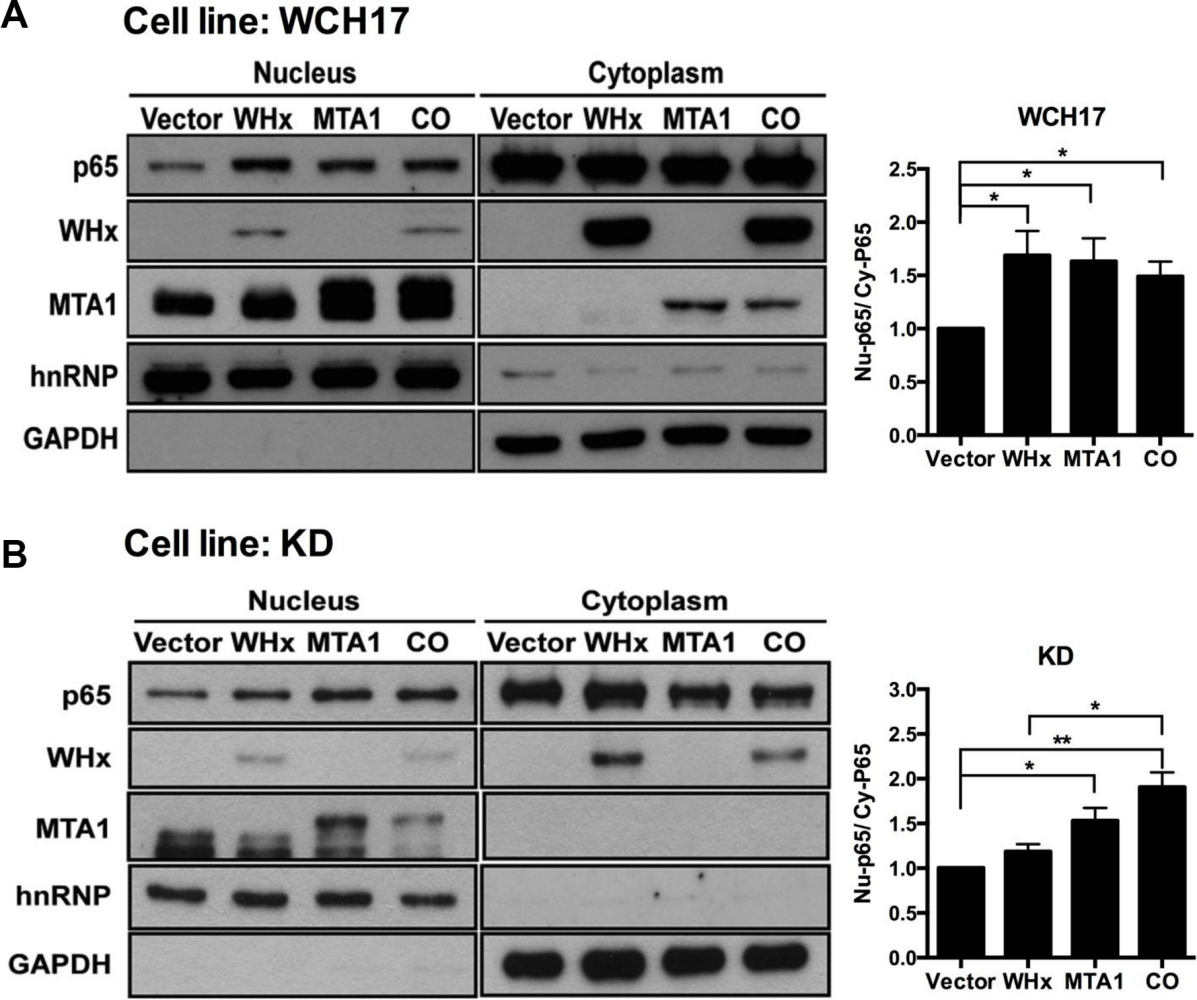
Wk-MTA1 plays an essential role in WHx-induced wk-NF-κB activation P65 nuclear translocation was assessed in WCH17 (**A**) and KD/wk-MTA1 WCH17 (designated as KD) (**B**) cells. The expression levels of wk-p65, WHx, and wk-MTA1 were determined using Western blotting. The relative ratio between nuclear (Nu) and cytoplasmic (Cy) wk-p65 was calculated using a densitometric analysis of the corresponding bands in Western blotting. Moreover, GAPDH and hnRNP served as markers for cytoplasmic and nuclear loading controls, respectively. CO: cells cotransfected with WHx and wk-MTA1-expressing plasmids (*P < .05; **P < .01).

Coimmunoprecipitation and Western blotting not only revealed that wk-MTA1 could interact with WHx (Figure 4, lanes 5 and 6) but also demonstrated that WHx and wk-MTA1 could form a complex with the p65 of NF-κB in 293T cells (Figure 4, bottom panel). Moreover, wk-MTA1 and WHx could activate NF-κB in WCH17 (Figure 5A).

To further examine whether wk-MTA1 plays a role in the WHx-mediated activation of wk-NF-κB, we established a wk-MTA1-knocked down WCH17 stable cell line (KD/wk-MTA1 WCH17), in which the expression level of wk-MTA1 was significantly decreased (Figure S4). Our result revealed that, although the expression level of WHx is comparable between parental and KD/wk-MTA1 WCH17 cell lines, WHx could significantly activate NF-κB and induce the expression of its downstream target metalloproteinase 9 (MMP-9) in parental cells but not in KD/wk-MTA1 cells (Figures 5A, 5B and S5). However, when ectopic expression resumed wk-MTA1 expression in KD/wk-MTA1 WCH17 cells, WHx could reinduce wk-p65 activation (CO vs WHx, Figures 5B and S5).

### Wk-MTA1 enhances tumor cell motility and invasiveness

The activation of NF-κB pathways by HBx is involved in promoting invasion and metastasis in HCC cells [21]. Therefore, we examined whether wk-MTA1 overexpression could also enhance WHx-mediated migration and invasion.

**Figure 6:**
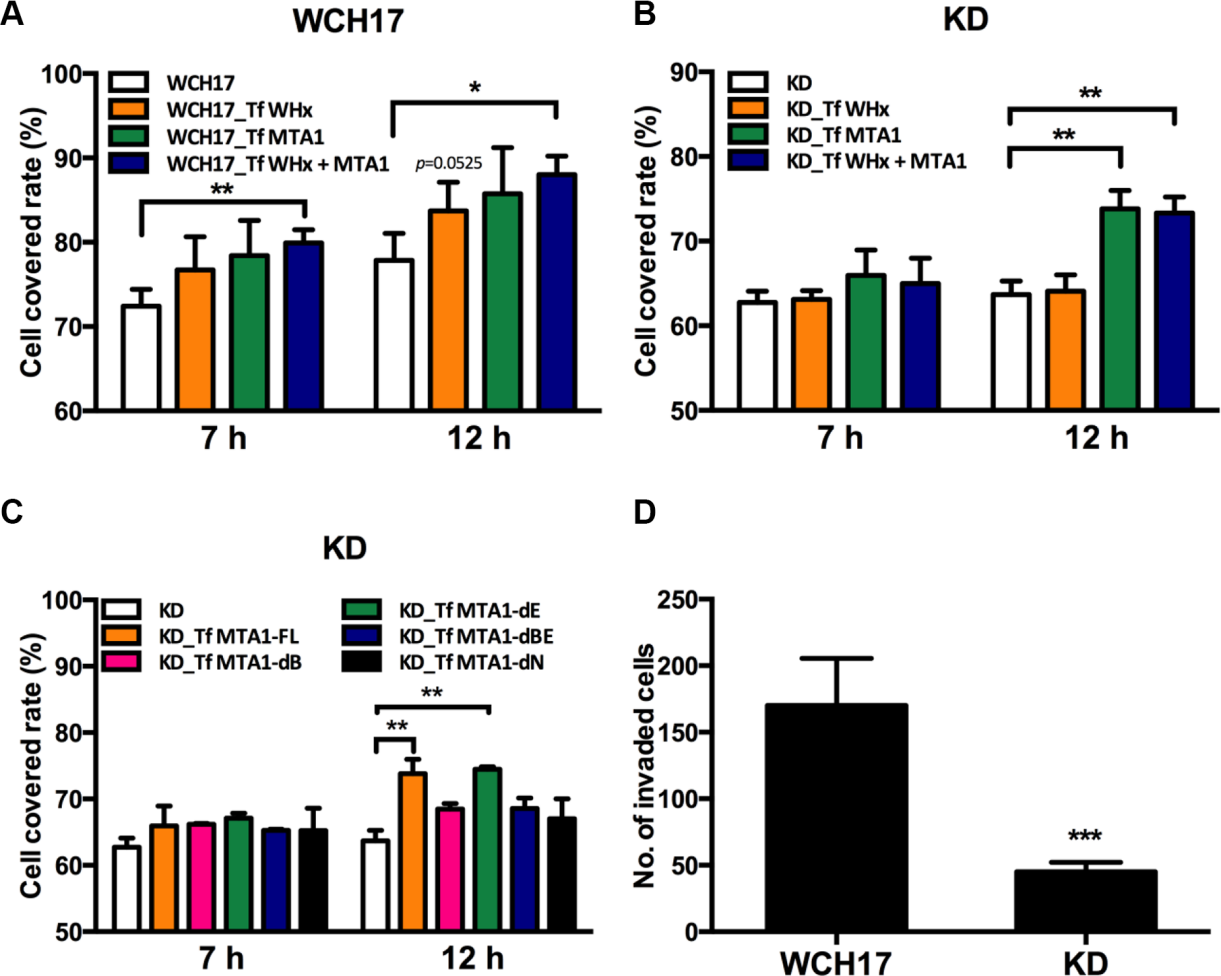
Wk-MTA1 enhances cell motility and promotes tumor cell invasiveness Wound healing assay was used for determining the migration ability of WCH17 (**A**), KD/wk-MTA1 WCH17 (designated as KD) (**B** and **C**) cells. Cells were transfected with vector, WHx, or MTA1-expressing plasmids. At 24 hours posttransfection, cells were harvested and replated for migration assays. The cell-covered area within the gap was measured at 0, 7, and 12 hours after reseeding. (C) KD cells were transfected with plasmids expressing different domains of MTA1 and cell mobility of these cells were determined by wound healing assay. (**D**) The invasiveness of WCH17 and KD cells was determined using the transwell assay. The number of invaded cells was quantified. All the experiments were presented as mean ± SD of 3 independent experiments (*P < .05, **P < .01 and ***P < .001). Representative photos of tumor cell migration and invasion are presented in Figures S8 and S9.

Using wound healing assay, we demonstrated that the overexpression of WHx and wk-MTA1 could promote the migration ability of WCH17 cells (Figure 6A). When wk-MTA1 expression was knocked down by short hairpin (sh)RNA in KD/wk-MTA1 WCH17 cells, the migration ability of the cells was significantly attenuated compared with that of parental WCH17 cells (Figure 6A and 6B). Notably, WHx lost its mobility-promoting activity in the absence of wk-MTA1. The resumption of wk-MTA1 expression in KD/wk-MTA1 WCH17 cells could rescue the WHx-mediated migration ability of the cells. These results suggested that wk-MTA1 is crucial for WHx-regulated HCC cell migration.

To define the critical domains responsible for the mobility-promoting activity of MTA1, we constructed a series of deletion mutants, in which different wk-MTA1 domains were deleted (Figure S1E). Through this approach, we observed that both BAH and NLS domains were crucial for the migration-promoting ability of MTA1. Without either of these domains, wk-MTA1 could not rescue the migration ability of KD/wk-MTA1 WCH17 cells (Figure 6C).

Using matrigel invasion assay, we revealed that wk-MTA1 knockdown reduced cell invasiveness in KD/wk-MTA1 WCH17 cells. The relative number of the invaded WCH17 cells [mean (± SD): 170.2 ± 15.77 cells] was approximately 3.8 times higher than that of KD/wk-MTA1 WCH17 cells [mean (± SD): 45 ± 7.09 cells; Figure 6D]. These results revealed that wk-MTA1 played a significant role in promoting HCC cell migration and invasion.

## DISCUSSION

MTA1 overexpression is highly associated with poor prognosis and tumor invasiveness in patients with HCC [11, 22]. Furthermore, MTA1 overexpression is often associated with HBV–HCC but seldom with HCV–HCC [11]; however, the underlying mechanisms remain elusive. An appropriate animal model of HBV–HCC could facilitate studying the molecular mechanisms underlying MTA1 overexpression in HBV–HCC and serve as a preclinical model for exploring the clinicopathological significance and potential clinical applications of MTA1. WHV-infected woodchucks have long been considered as one of the most appropriate animal models of HBV-associated diseases, including HCC [23–25]. Similar to HBV–HCC in humans, HCC spontaneously developed in woodchucks because of a chronic WHV infection. Furthermore, woodchuck HCC typically develops within 1–4 years after a WHV infection [23], and the progression of HBV–HCC can be monitored within a reasonable time frame. Therefore, WHV-induced HCC-bearing woodchucks are more biologically relevant model than small rodents for HBV–HCC. In this study, we investigated the expression pattern, spliced variants, cell migration- and invasion-promoting activities, and molecular functions of wk-MTA1 in WHV-induced HCC for determining the translational value of this model in studying the function of MTA1 in HBV–HCC.

To characterize the expression and function of wk-MTA1 in the woodchuck HCC model, we first cloned the cDNA of wk-MTA1. We observed remarkable conservation of nucleotide and protein sequences of MTA1 among different species, indicating that MTA1 is a highly conserved protein and may have critical biological functions in evolution. Moreover, wk-MTA1, similar to human MTA1, was overexpressed in woodchuck primary HCCs. We observed wk-MTA1dE4 overexpression in the tumor tissues of most woodchuck HCC models, accounting for approximately 50% of the total wk-MTA1. MTA1dE4 appeared to be a conserved spliced variant among different species and was well detected in both humans and mice. The percentage of MTA1-overexpressed WHV-induced HCC increased from 61.1% to 77.8% when wk-MTA1dE4 instead of the total wk-MTA1 was assayed. In addition, overexpression of wk-MTA1dE4 was found to be associated with all tumor types, including the larger tumors. In contrast, the association between total wk-MTA1 with single type and larger tumors could not be established. Due to the small sample size for these two types of tumors, we could not exclude the possible correlation between wk-MTA1dE4 and single tumor type in the woodchuck HCC models currently. Based on these data, we found that wk-MTA1dE4 may represent a more sensitive marker than total wk-MTA1 in woodchuck HCC. The differential roles of full-length MTA1 and its spliced variants in oncogenesis have been well documented for some proteins [9, 26, 27]. MTA1s, another MTA1 spliced variant, but not full-length MTA1, has been reported to sequester the estrogen receptor in the cytoplasm and contribute to poor prognosis of breast tumors [26, 28, 29]. However, the molecular functions of wk-MTA1dE4 were previously unexplored. As per our review of relevant literature, our study of the woodchuck model is the first to reveal that wk-MTA1dE4 may play a critical role in hepadnavirus-induced HCC. Previous studies of human clinical samples have not examined whether the overexpressed MTA1 is a full-length protein or a spliced variant. Therefore, it was crucial to assess whether the findings obtained in the woodchuck model can be validated in clinical samples and whether they can address the biological and clinical prominence of MTA1dE4 in HCC.

Woodchuck MTA1 not only exhibited a similar overexpression pattern in hepadnavirus-induced HCC but also revealed molecular characteristics similar to those of human MTA1 with respect to critical roles of MTA1 in hepadnavirus X protein-related hepatocarcinogenesis. The results of the woodchuck model were analogous to those of the human HCC cell line. Both HBx and WHx could induce MTA1 expression and interact with the corresponding MTA1 protein [17, 19]. In addition, both human and woodchuckMTA1 were essential for the X protein-mediated NF-κB activation [7, 19]. We also demonstrated that wk-MTA1 played a significant role in WHx-mediated tumor cell invasion and promotion, a function that had not previously been studied in the human HCC cell model. We suggest that WHx increases the migratory activity of parental WCH17 cells mainly by upregulating the expression level of endogenous wk-MTA1. In parental cells, the amount of wk-MTA1 is abundant enough to enhance the cell mobility. Therefore, the effect of WHx and ectopic wk-MTA1 on further increasing migratory ability of WCH17 cells is not apparent (Figure 6A). In KD cells, WHx induced endogenous wk-MTA1 expression was efficiently suppressed by the presence of shRNA and only the ectopic expressed wk-MTA1, shRNA resistant wk-MTA1, could function in enhancing cell mobility. Therefore, co-transfection of WHx and wk-MTA1 cannot further enhance KD cell migration compared with wk-MTA1 transfection alone (Figure 6B). Our study here revealed the potential mechanisms underlying MTA1 overexpression in HBV–HCC with a higher degree of invasiveness; the results also support the notion that WHV-infected woodchucks represent an appropriate preclinical model for studying the biological functions and clinical significance of MTA1 in HBV–HCC.

Notably, the percentage of MTA1 overexpression was higher in WHV-induced HCCs (61.1%) than in human HBV–HCC (34.3%) [30]. Previous transcriptomic analyses have revealed that WHV-induced HCC is positively correlated with the S2 subclass of human HCC, which is associated with MYC activation [25]. The *MTA1* gene was a target of the c-MYC oncoprotein, and MTA1 was essential for the oncogenic function of c-MYC [31]. Therefore, the percentage of wk-MTA1 overexpression in woodchuck HCC was higher possibly because of the nature of WHV-induced HCC; that is, more than half of WHV-induced HCC are involved in activating N-myc2, a member of the myc family [32]. We observed a positive correlation between the expression levels of wk-*MTA1* and N-myc*2* in woodchuck HCC tissues (Figure S6). Thus, MTA1 overexpression may have been associated with an HCC subtype (eg, HCCs with S2 signatures). The clinical features reported for MTA1-overexpressed human HCC revealed a high similarity to the characteristics of the S2 subclass, including large tumor, poor differentiation, early recurrence, and poor survival. Further studies are required for examining whether MTA1-overexpressed human HCCs exhibit molecular signatures of the S2 subclass. Furthermore, examining the role of MTA1 in driving hepatocarcinogenesis in the MYC-activated pathway would be an interesting topic worth exploring.

The present woodchuck HCC model has limitations. The lack of an entire genome sequence and appropriate materials impede exploring the molecular mechanisms underlying hepatocarcinogenesis. However, the recent availability of transcriptome data has partially resolve this obstacle and facilitated the molecular study of this model [25]. Another potential limitation is that the molecular mechanisms underlying hepatocarcinogenesis in woodchuck HCC may not represent all types of human HCCs. However, our results indicate that woodchuck HCC resembles human HCC at least in the molecular functions of MTA1 in hepadnavirus-induced hepatocarcinogenesis. Therefore, the results yielded by the WHV-infected woodchuck model could be extrapolated and applied to human MTA1-overexpressed HCC.

In conclusion, we demonstrated the translational value of the woodchuck model and characterized the molecular functions of MTA1 in hepadnavirus-induced HCC through an interaction with the viral X protein. Thus, the WHV-infected woodchuck model can be used for addressing specific and critical questions that cannot be completely explored in humans. Our study also provided clues on the potential clinical significance of the MTA1-spliced variant in hepatocarcinogenesis. Overall, this study lays the foundation for further studies exploring the functions of MTA1 in HBV–HCC in the woodchuck model and humans.

## MATERIALS AND METHODS

### Experimental design

In this study, we used the woodchuck HCC model and *in vitro* culture system for exploring the role of MTA1 in HBV–HCC. A detailed description of the experimental procedures is provided in Figure S7.

### Animals

Woodchucks (*Marmota monax*) were purchased from North Eastern Wildlife (Ithaca, NY, USA) and were maintained at the laboratory animal center, National Taiwan University College of Medicine. Experiments were conducted in accordance with the protocols approved by the Animal Care and Use committee of National Taiwan University College of Medicine. Autopsy liver tissues of woodchucks were snap frozen in liquid nitrogen and stored at −80°C until RNA extraction.

### Extraction of RNAs from a woodchuck hepatoma cell line and liver tissues

The total RNA was extracted from 50 mg of woodchuck liver tumor and nontumor tissues and from 4 × 10^6^ cells of WCH17 by using Trizol (Invitrogen Life Technologies; CA, USA) or Totally RNA kit (Ambion; TX, USA) according to the manufacturers' instructions.

### Cloning of wk-MTA1

We used PCR-based strategies, including RLM-RACE and conventional PCR, for cloning the full-length wk-MTA1. The sequence of wk-MTA1 was unavailable; therefore, MTA1-specific primers were designed on the basis of the highly conserved region of known MTA1 sequences from other species [humans (accession: NM_004689.3), chimpanzees (accession: XR_023693.2), mice (accession: NM_054081.2), rats (accession: NM_022588.1), horses (accession: XM_001915037.1), dogs (accession: XM_537568.2), rhesus monkeys (accession: XM_002808471.1), and white tufted-ear marmosets (accession: XM_002754333.1)].

cDNA was synthesized from 1 μg of ligated RNA by using ThermoScript™ RNase H^−^ reverse transcriptase (Invitrogen Life Technologies; CA, USA) with oligo (dT) primers and random hexamers. The resultant cDNA was then used as the template for amplifying 3 fragments of wk-MTA1 cDNA to assemble the full-length wk-MTA1 cDNA (Figure S1A). The sequences of primers and details of PCR amplification programs are listed in Tables S1 and S2, respectively. Moreover, the locations of the primers relative to MTA1 are illustrated in Figure S1A. The sequence of wk-MTA1 was analyzed for similarity with that of MTA1 from other species by using the BLASTN program of the National Center for Biotechnology Information.

### Plasmid construction

We reverse transcribed 0.5 μg of the total RNA from WCH17 cells into cDNA by using the Superscript III first-strand synthesis system (Invitrogen; San Diego, CA, USA). The full-length cDNA of wk-MTA1 was obtained by PCR amplifying the cDNA with the primers MTA1-FC and MTA1-RC (Table S1) and was then cloned into the EcoRI and XbaI sites of the expression vector p3xFlag-CMV10 (Sigma–Aldrich; MO, USA), resulting in the plasmid wk-MTA1/p3xFlag-CMV10. Furthermore, we performed site-directed mutagenesis for constructing an shRNA-resistant construct, wk-MTA1(ES)/p3xFlag-CMV10, in which the target sequence of the MTA1 shRNA (pLKO–shMTA1-1) was mutated without changing the amino acid sequence.

shRNA-resistant wk-MTA1 deletion mutants were constructed using the In-Fusion^®^ HD cloning kit (Clontech; Mountain View, CA, USA). MTA1-dB, MTA1-dE, MTA1-dBE, and MTA1-dN designated wk-MTA1 expression plasmids as bromoadjacent homology (BAH), Egl-27 and MTA1 homology 2 (ELM2), BAH plus ELM2, and all nucleotides after ELM2 as deleted, respectively (the maps of these constructs are presented in Figure S1E). The nucleotide sequences of all constructs were verified through sequencing.

### Analysis of wk-MTA1 RNA using northern blotting

We separated 15 μg of the total RNA through electrophoresis by using a 1.2% agarose gel, which was then transferred onto a positively charged nylon membrane (Roche Diagnostics; Mannheim, Germany). wk-MTA1 mRNA was detected using a digoxigenin (DIG)-labeled wk-MTA1 probe (spanning nt +392 to +910) generated using the DIG oligonucleotide tailing kit (Roche Diagnostics). Moreover, 18S rRNA was used as the loading control and was detected using a wk-18S rRNA-specific probe.

### Quantitative RT-PCR

The wk-MTA1 mRNA expression level in woodchuck liver tissues was determined using qRT-PCR with the primers MTA1-FQ and MTA1-RQ by using the SteponeTM Real-Time PCR system (Applied Biosystems; Foster, CA, USA) with the following thermal profile: 40 cycles of 10-second denaturation at 95°C, 10-second annealing at 60°C, and 10-second extension at 72°C. The detailed primer sequences are listed in Table S1. The expression level was calculated using interpolation against a standard curve generated with a plasmid containing the target sequence. The expression level of DDX5, used as a internal control, was also determined. Melting curve analysis was performed to ensure the specificity of the PCR product.

### Cell culture and transfection

WCH17 (ATCC^®^ CRL-2082™) and HEK-293T cells were maintained in Dulbecco's modified Eagle's medium (Invitrogen Life Technologies; CA, USA) supplemented with 10% fetal bovine serum (FBS; Biological Industries; Israel) at 37°C in a humidified chamber with 5% CO_2_. The cells were transfected at a cell density of 3.5 × 10^5^ cells per well in 6-well plates or 4 × 10^6^ cells per 10-cm dish. Transient transfections were performed using Lipofectamine 2000 (Invitrogen Life Technologies; CA, USA) according to the manufacturer's instructions.

### Establishment of stable cell lines

To generate a KD/wk-MTA1 WCH17 cell line, a shRNA lentiviral expression system was used. shRNA-expressing vectors, pLKO-shMTA1-1 (Clone ID: TRCN0000230497; target sequence 5′-TGCGCATCTTGTTGGACATAT-3′) and pLKO-sh-mMTA1 (Clone ID: TRCN0000039261; target sequence: 5′-CCGCAGGA TTGAAGAGCTTAA-3′) were obtained from the National RNAi Core Facility (Institute of Molecular Biology, Academia Sinica; Taipei, Taiwan). shRNA expressed by pLKO–shMTA1-1 could effectively suppress wk-MTA1 expression, whereas shRNA expressed by pLKO–sh-mMTA1 could knock down mouse MTA1 but not woodchuck MTA1 expression and was used as the negative control. Moreover, recombinant lentiviruses expressing shRNA were produced as previously described [33]. WCH17 cells were transduced with recombinant lentiviruses, and stable cell lines were selected with 1 μg/mL puromycin. The puromycin-resistant cells stably expressing shMTA1-1 and sh-mMTA1 were called KD/wk-MTA1 and KD/CTL, respectively.

### Antibodies

The following antibodies were used in this study: antibodies against MTA1 (sc-9446) and hnRNP (sc-10045) were purchased from Santa Cruz Biotechnology (CA, USA); the NF-kB P65 antibody (8242) and phosphorylated p65 antibody (3033) were purchased from Cell Signaling (MA, USA); the MMP-9 antibody (MA5-15886) was purchased from Thermo Scientific (MA, USA). Moreover, the flag-M2 monoclonal antibody (F3165) was purchased from Sigma Chemical Co. (St. Louis, MO, USA); the GAPDH antibody (PA1-16777) and GFP monoclonal antibody (632381) were purchased from Pierce (IL, USA) and Clontech (CA, USA), respectively.

### Extraction of nuclear and cytoplasmic proteins

In brief, the cells were differentially lysed using a nuclear extract buffer [20 mM Tris Cl, 420 mM NaCl, 1.5 mM MgCl_2_, 0.2 mM EDTA, 1 mM PMSF, and 25% (v/v) glycerol; pH 8.0] and cytoplasmic extract buffer [10 mM HEPES, 60 mM KCl, 1 mM EDTA, 0.075% (v/v) NP40, 1 mM DTT, and 1 mM PMSF; pH 7.6]. Proteins in different fractions were analyzed using Western blotting with specific antibodies. HnRNP and GAPDH were used as nuclear and cytoplasmic loading controls, respectively. The signals were visualized using the enhanced chemiluminescence kit (Roche; Germany). Densitometric analyses of the bands were performed using Alphaimage 2000.

### Coimmunoprecipitation

In brief, cultured cells were lysed with RIPA lysis buffer containing protease inhibitor (120 mM NaCl, 50 mM Tris pH 7.5, 5 mM EDTA, 0.5% NP-40, and 1 mM freshly prepared PMSF). Cell lysates were immunoprecipitated with specific antibodies overnight at 4°C, and the immunocomplexes were then captured using protein-G sepharose (Amersham Pharmacia Biotech AB; Sweden) with gentle inverting at 4°C for 2 hours. Subsequently, immunocomplexes were analyzed using Western blotting with specific antibodies.

### Immunohistochemistry

IHC staining for wk-MTA1 was performed using the streptavidin-biotinylated immunoenzymatic antigen detection system (Spring Bioscience; USA) according to the manufacturer's instructions. The 4-μm thick sections were obtained from formalin-fixed, paraffin-embedded tissue blocks. The sections were incubated with MTA1 antibody at 4°C overnight. The peroxidase activity was detected using the enzyme substrate 3′-diaminobenzidine, and the section was counterstained with Gill's hematoxylin (Polysciences; Germany).

### Wound healing and transwell assays

Migration assays using culture inserts with gaps of 500 μm (Ibidi; Martinsried, Germany) were performed according to the manufacturer's instructions. Cells were seeded on 6-cm dishes, transfected with different plasmids for 24 hours, plated at 4.9 × 10^4^ cells/insert, and grown overnight to form a monolayer with a gap. The inserts were subsequently removed, and the residual gap area was imaged and measured at 0, 7, and 12 hours after the insert was removed to quantify the cell migration distance. Each experiment was performed independently at least 3 times.

The invasiveness of various cells was examined using the transwell invasion assay with BD Biocoat Matrigel Invasion Chambers (BD Biosciences; Bedford, MA, USA), as previously described [34]. Moreover, 5 × 10^4^ cells in a serum-free medium were added to the top inserts. The bottom chambers were filled with a medium containing 10% FBS, which served as a chemoattractant. After 22 hours, the noninvaded cells were removed, and cells on the lower surface were fixed and stained with hematoxylin and eosin. The numbers of invaded cells were determined by imaging them in 5 random fields per insert at 200× magnification.

### Statistical analysis

The statistical significance of differences in mean values was assessed through a Student *t* test and one-way repeated measure ANOVA by using GraphPad Prism5 (GraphPad Software; CA, USA) and SPSS 16.0 statistical software. *P* < .05 was considered statistically significant. Average values were expressed as mean ± SD.

## SUPPLEMENTARY MATERIALS FIGURES AND TABLES



## References

[1] el-Serag HB (2001). Epidemiology of hepatocellular carcinoma. Clin Liver Dis.

[2] Parkin DM, Stjernsward J, Muir CS (1984). Estimates of the worldwide frequency of twelve major cancers. Bulletin of the World Health Organization.

[3] Matsumata T, Kanematsu T, Shirabe K, Sonoda T, Furuta T, Sugimachi K (1990). Decreased morbidity and mortality rates in surgical patients with hepatocellular carcinoma. Br J Surg.

[4] Inagawa S, Itabashi M, Adachi S, Kawamoto T, Hori M, Shimazaki J, Yoshimi F, Fukao K (2002). Expression and prognostic roles of beta-catenin in hepatocellular carcinoma: correlation with tumor progression and postoperative survival. Clinical cancer research.

[5] Buurman R, Gurlevik E, Schaffer V, Eilers M, Sandbothe M, Kreipe H, Wilkens L, Schlegelberger B, Kuhnel F, Skawran B (2012). Histone deacetylases activate hepatocyte growth factor signaling by repressing microRNA-449 in hepatocellular carcinoma cells. Gastroenterology.

[6] Moeini A, Cornella H, Villanueva A (2012). Emerging signaling pathways in hepatocellular carcinoma. Liver cancer.

[7] Li DQ, Kumar R (2015). Unravelling the Complexity and Functions of MTA Coregulators in Human Cancer. Adv Cancer Res.

[8] Nicolson GL, Nawa A, Toh Y, Taniguchi S, Nishimori K, Moustafa A (2003). Tumor metastasis-associated human MTA1 gene and its MTA1 protein product: role in epithelial cancer cell invasion proliferation and nuclear regulation. Clin Exp Metastasis.

[9] Toh Y, Nicolson GL (2009). The role of the MTA family and their encoded proteins in human cancers: molecular functions and clinical implications. Clin Exp Metastasis.

[10] Toh Y, Nicolson GL (2014). Properties and clinical relevance of MTA1 protein in human cancer. Cancer Metastasis Rev.

[11] Ryu SH, Chung YH, Lee H, Kim JA, Shin HD, Min HJ, Seo DD, Jang MK, Yu E, Kim KW (2008). Metastatic tumor antigen 1 is closely associated with frequent postoperative recurrence and poor survival in patients with hepatocellular carcinoma. Hepatology.

[12] Hamatsu T, Rikimaru T, Yamashita Y, Aishima S, Tanaka S, Shirabe K, Shimada M, Toh Y, Sugimachi K (2003). The role of MTA1 gene expression in human hepatocellular carcinoma. Oncol Rep.

[13] Lin CL, Kao JH (2013). Risk stratification for hepatitis B virus related hepatocellular carcinoma. Journal of gastroenterology and hepatology.

[14] Beasley RP, Hwang LY, Lin CC, Chien CS (1981). Hepatocellular carcinoma and hepatitis B virus. A prospective study of 22 707 men in Taiwan. Lancet.

[15] Michielsen PP, Francque SM, van Dongen JL (2005). Viral hepatitis and hepatocellular carcinoma. World J Surg Oncol.

[16] Park NH, Song IH, Chung YH (2007). Molecular Pathogenesis of Hepatitis-B-virus-associated Hepatocellular Carcinoma. Gut Liver.

[17] Bui-Nguyen TM, Pakala SB, Sirigiri DR, Martin E, Murad F, Kumar R (2010). Stimulation of inducible nitric oxide by hepatitis B virus transactivator protein HBx requires MTA1 coregulator. J Biol Chem.

[18] Yoo YG, Na TY, Seo HW, Seong JK, Park CK, Shin YK, Lee MO (2008). Hepatitis B virus X protein induces the expression of MTA1 and HDAC1 which enhances hypoxia signaling in hepatocellular carcinoma cells. Oncogene.

[19] Bui-Nguyen TM, Pakala SB, Sirigiri RD, Xia W, Hung MC, Sarin SK, Kumar V, Slagle BL, Kumar R (2010). NF-kappaB signaling mediates the induction of MTA1 by hepatitis B virus transactivator protein HBx. Oncogene.

[20] Ng SA, Lee C (2011). Hepatitis B virus X gene and hepatocarcinogenesis. J Gastroenterol.

[21] Zhang X, You X, Wang Q, Zhang T, Du Y, Lv N, Zhang Z, Zhang S, Shan C, Ye L, Zhang X (2012). Hepatitis B virus X protein drives multiple cross-talk cascade loops involving NF-kappaB 5-LOX OPN and Capn4 to promote cell migration. PLoS One.

[22] Moon WS, Chang K, Tarnawski AS (2004). Overexpression of metastatic tumor antigen 1 in hepatocellular carcinoma: Relationship to vascular invasion and estrogen receptor-alpha. Hum Pathol.

[23] Tennant BC (2001). Animal models of hepadnavirus-associated hepatocellular carcinoma. Clin Liver Dis.

[24] Tennant BC, Toshkov IA, Peek SF, Jacob JR, Menne S, Hornbuckle WE, Schinazi RD, Korba BE, Cote PJ, Gerin JL (2004). Hepatocellular carcinoma in the woodchuck model of hepatitis B virus infection. Gastroenterology.

[25] Fletcher SP, Chin DJ, Ji Y, Iniguez AL, Taillon B, Swinney DC, Ravindran P, Cheng DT, Bitter H, Lopatin U, Ma H, Klumpp K, Menne S (2012). Transcriptomic analysis of the woodchuck model of chronic hepatitis B. Hepatology.

[26] Kumar R, Wang RA, Mazumdar A, Talukder AH, Mandal M, Yang Z, Bagheri-Yarmand R, Sahin A, Hortobagyi G, Adam L, Barnes CJ, Vadlamudi RK (2002). A naturally occurring MTA1 variant sequesters oestrogen receptor-alpha in the cytoplasm. Nature.

[27] Li DQ, Pakala SB, Nair SS, Eswaran J, Kumar R (2012). Metastasis-associated protein 1/nucleosome remodeling and histone deacetylase complex in cancer. Cancer Res.

[28] Mishra SK, Yang Z, Mazumdar A, Talukder AH, Larose L, Kumar R (2004). Metastatic tumor antigen 1 short form (MTA1s) associates with casein kinase I-gamma2 an estrogen-responsive kinase. Oncogene.

[29] Manavathi B, Kumar R (2007). Metastasis tumor antigens an emerging family of multifaceted master coregulators. J Biol Chem.

[30] Jin YJ, Chung YH, Kim JA, Park WH, Lee D, Seo DD, Ryu SH, Jang MK, Yu E, Lee YJ (2012). Factors predisposing metastatic tumor antigen 1 overexpression in hepatitis B virus associated hepatocellular carcinoma. Digestive diseases and sciences.

[31] Zhang XY, DeSalle LM, Patel JH, Capobianco AJ, Yu D, Thomas-Tikhonenko A, McMahon SB (2005). Metastasis-associated protein 1 (MTA1) is an essential downstream effector of the c-MYC oncoprotein. Proc Natl Acad Sci U S A.

[32] Hansen LJ, Tennant BC, Seeger C, Ganem D (1993). Differential activation of myc gene family members in hepatic carcinogenesis by closely related hepatitis B viruses. Mol Cell Biol.

[33] Hung SC, Wu IH, Hsue SS, Liao CH, Wang HC, Chuang PH, Sung SY, Hsieh CL (2010). Targeting l1 cell adhesion molecule using lentivirus-mediated short hairpin RNA interference reverses aggressiveness of oral squamous cell carcinoma. Mol Pharm.

[34] Yao J, Liang L, Huang S, Ding J, Tan N, Zhao Y, Yan M, Ge C, Zhang Z, Chen T, Wan D, Yao M, Li J (2010). MicroRNA-30d promotes tumor invasion and metastasis by targeting Galphai2 in hepatocellular carcinoma. Hepatology.

[35] Huang KW, Wu HL, Lin HL, Liang PC, Chen PJ, Chen SH, Lee HI, Su PY, Wu WH, Lee PH, Hwang LH, Chen DS (2010). Combining antiangiogenic therapy with immunotherapy exerts better therapeutical effects on large tumors in a woodchuck hepatoma model. Proc Natl Acad Sci U S A.

