# Supplementary material for: Characterization of metastatic tumor antigen 1 and its interaction with hepatitis B virus X protein in NF-κB signaling and tumor progression in a woodchuck hepatocellular carcinoma model
Source: Oncotarget. 2016 Jun 13;7(30):47173–85. doi: 10.18632/oncotarget.9986 (PMC5216933; doi:10.18632/oncotarget.9986)
Supplement: Supplementary file 1 [file oncotarget-07-47173-s001.pdf]

Characterization of metastatic tumor antigen 1 and its interaction with hepatitis B virus X protein in NF-κB signaling and tumor progression in a woodchuck hepatocellular carcinoma model

Supplementary Materials

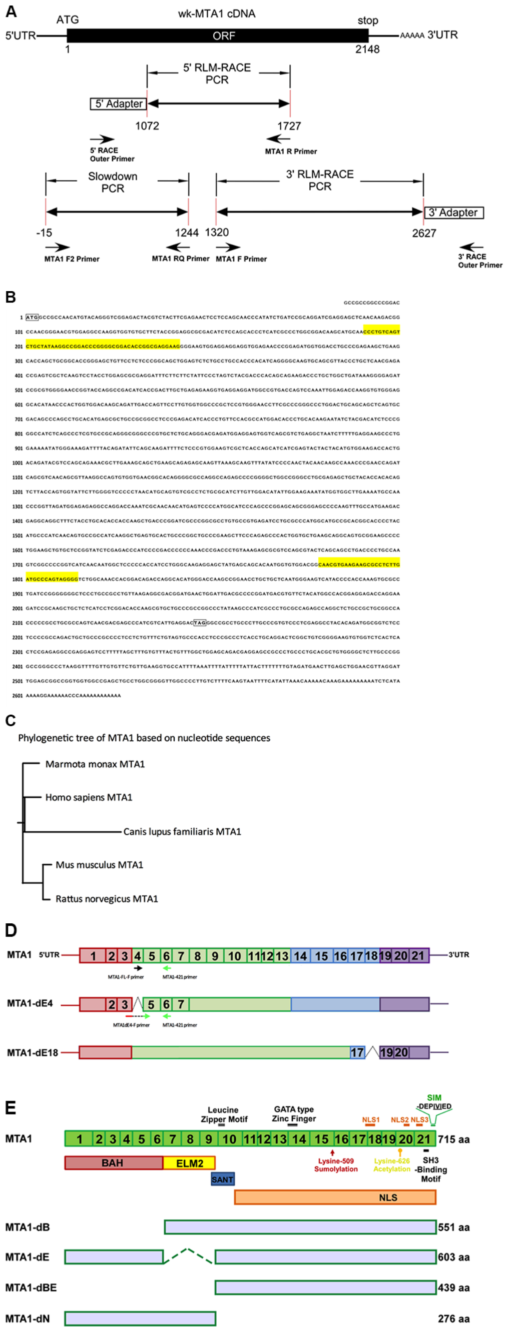

Supplementary Figure S1: Illustration of wk-MTA1 cloning strategies, cDNA sequence, and organization of protein domains. Cloning strategies of woodchuck MTA1 cDNA (**A**) and its cDNA sequence (**B**). The nucleotides for the start and stop codons are boxed and bold-faced, respectively. Two alternative splice variants of wk-MTA1 used in this study are shaded in yellow. (**C**) Phylogenetic analysis for MTA1 nucleotide sequences among different species. (**D**) The schematic representation of the full-length wk-MTA1 and its 2 spliced variants wk-MTA1dE4 and wk-MTA1dE18. The broken line indicates the splicing events of wk-MTA1. Moreover, arrows indicate the region of sequence-specific primer pairs designed for distinguishing between wk-MTA1-FL and wk-MTA1dE4. (**E**) The map of wk-MTA1 and 4 deletion constructs.

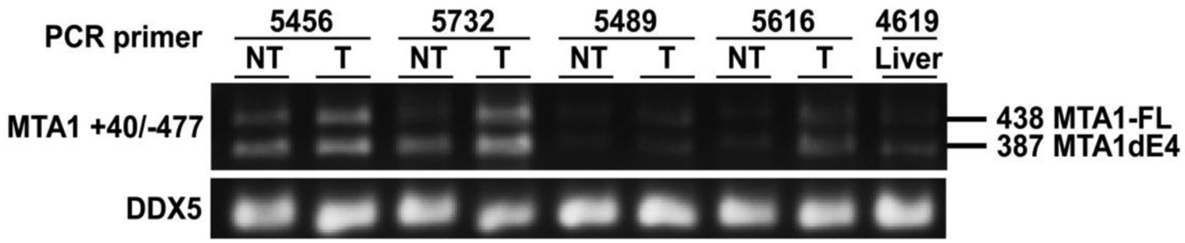

Supplementary Figure S2: Presence of wk-MTA1 spliced variant wk-MTA1dE4 was examined in woodchuck autopsy samples. The expression levels of wk-MTA1-FL and wk-MTA1dE4 were determined using conventional RT-PCR with primer pairs covering the spliced region of wk-MTAdE4 (MTA1, +40 and MTA1, −477) in liver tissues from one WHV-resolved woodchuck (designated as 4619 liver) and 4 woodchucks with paired tumor and nontumor tissues. DDX5 was used as the internal control.

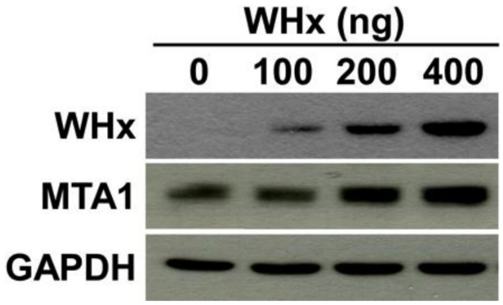

Supplementary Figure S3: WHx dose-dependently upregulates wk-MTA1 expression in WCH17 cells. Western blotting of WHx and wk-MTA1 in WCH17 cells transfected with either control vector (0) or WHx-expressing plasmids at different concentrations (100, 200, and 400 ng/transfection). GAPDH was used as the loading control.

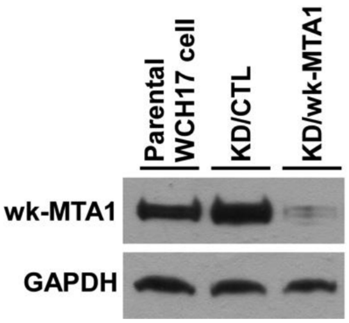

Supplementary Figure S4: Wk-MTA1 protein was significantly decreased in KD/wk-MTA1 cells but not in KD/CTL cells. WCH17 cells (designated as parental cells) were transfected with wk-MTA1-targeting shRNA (sh-MTA1-1) and control shRNA (sh-m-MTA1). Puromycin-resistant cells stably expressing shMTA1-1 and sh-mMTA1 were designated as KD/wk-MTA1 and KD/CTL (control designated as CTL). Cells were lysed, and the expression level of wk-MTA1 was examined using Western blotting.

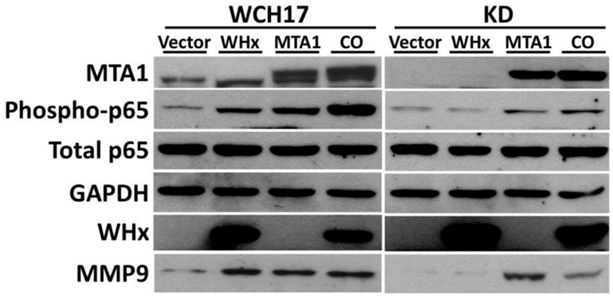

Supplementary Figure S5: Wk-MTA1 is essential for WHx-mediated NF-κB signaling. The expression levels of wk-MTA1, phosphorylated p65, total p65, WHx, and MMP9 were assessed in WCH17 and KD/wk-MTA1 WCH17 (designated as KD) cells by western blot. GAPDH served as a loading control. CO: cells cotransfected with WHx and wk-MTA1-expressing plasmids.

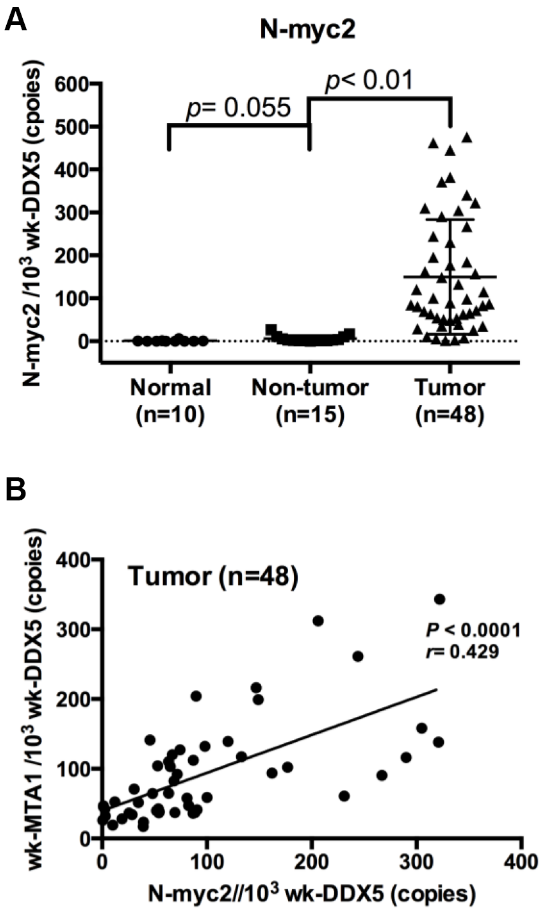

Supplementary Figure S6: N-myc2 was upregulated in woodchuck HCC tissues and positively correlated with the levels of wk-MTA1. (**A**) The expression of N-myc2 was detected using qRT-PCR with primer pairs (N-myc2, +780 and N-myc2, −1368) in normal and nontumor and tumor tissues. (**B**) A positive correlation was observed between wk-MTA1 expression and N-myc2 expression in woodchuck tumor liver samples (n = 48). The statistical significance and correlation coefficient r was determined using Pearson correlation. The expression of each target gene was normalized to 10^3 wk-DDX5 mRNA. The results are presented as mean ± SD.

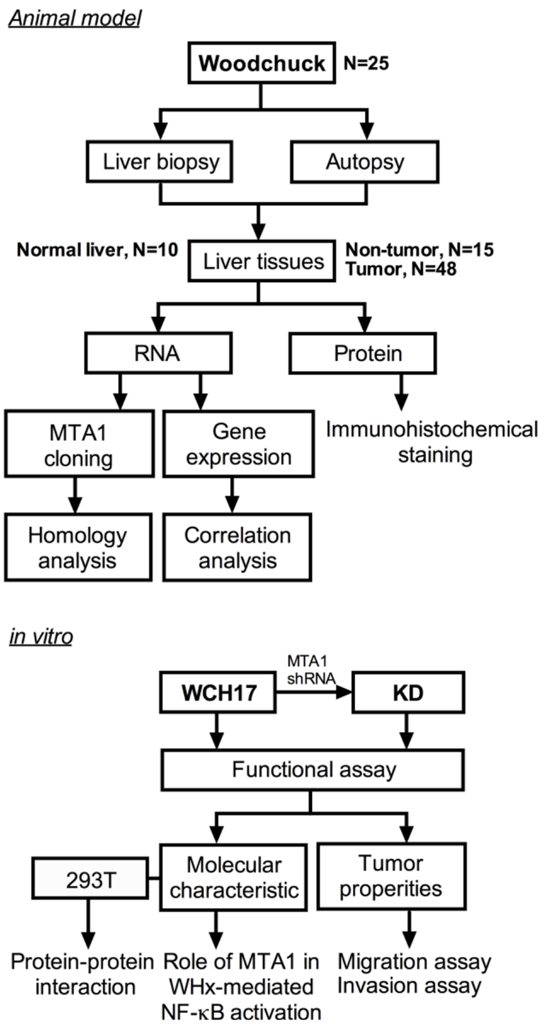

Supplementary Figure S7: Flowchart of the experimental approaches used in this study. Liver biopsies from 10 WHV-resolved woodchucks were designated as normal liver tissues. All paired tumor and nontumor tissues were obtained from 15 woodchucks. Four of the woodchucks had a single tumor, whereas the remaining woodchucks had multiple tumors.

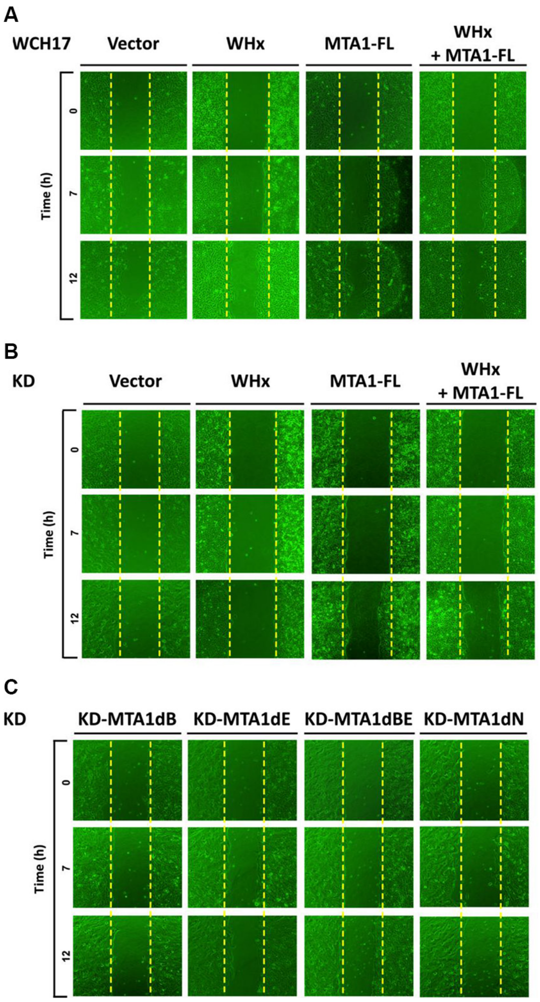

Supplementary Figure S8: Wound healing assay was employed to determine the migration ability of WCH17 and MTA1-knocked down cells. (**A**) WCH17 and (**B**) KD cells were transfected with an empty vector, WHx, and wk-MTA1 expression plasmids. (**C**) KD cells were transfected with wk-MTA1 expressing plasmids with different domain deletions. Cells were monitored and photographed at 7 and 12 hours after incubation. Original magnification: ×200.

A WCH17

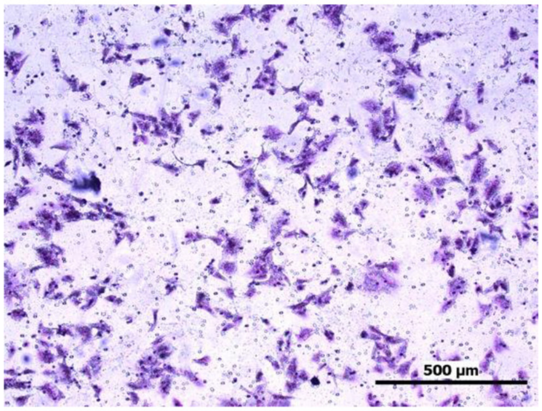

B KD

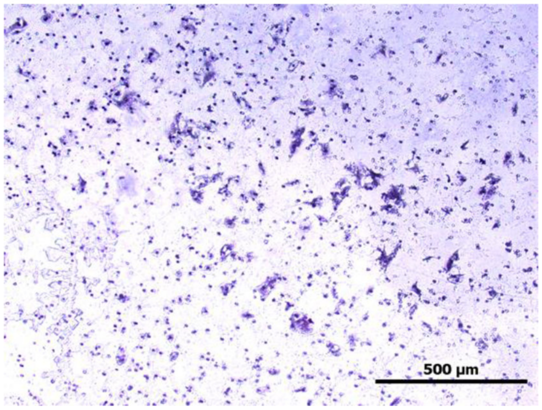

Supplementary Figure S9: Knockdown of wk-MTA1 expression reduces cell invasion ability. The invasiveness of WCH17 and KD cells was determined using the transwell assay. The invaded (**A**) WCH17 and (**B**) KD cells were photographed at 22 hours after plating. Original magnification: ×400.

Supplementary Table S1: Primers and probes used for cloning, Northern blotting, RT-PCR, and qRT-PCR analysis

Primer		Priming site[2]	Oligonucleotide sequence (5′→3′)
3′RACE Outer[1]	Rv		GCG AGC ACA GAA TTA ATA CGA CT
5′RACE Outer[1]	Fw		GCT GAT GGC GAT GAA TGA ACA CTG
5′RACE Inner[1]	Fw		CGC GGA TCC GAA CAC TGC GTT TGC TGG CTT TGA TGA AA
MTA1-F	Fw	1320→1346	GCC RGG ACC AAA CCG CAR TAA CAT GAG[3]
MTA1-R	Rv	1727→1710	GGA GCC ATT GTT GAT GAC C
MTA1-F2	Fw	−15→3	GCC GCC GGC CCG GAC ATG
MTA1-FC	Fw	1→12	G**GA ATT C**CG ACA TGG CCG CCA AC
MTA1-RC	Rv	2148→2135	GC**T CTA GA**G CCT AGT CCT CRA TGA[3]
MTA1 +392	Fw	392→414	TCA ACG AGA CCG AGT CGC TCA AG
MTA1 -910	Rv	910→888	CAT ATT TTT CCA GGG CTT CCT C
18 s rRNA F	Fw		CCC TGT AAT TGG AAT GAG TCC A
18 s rRNA R	Rv		ATC TGA TCG TCT TCG AAC CTC C
MTA1 +392<br>MTA1 -910<br>18 s rRNA F<br>18 s rRNA R	Fw<br>Rv<br>Fw<br>Rv	392→414<br>910→888	TCA ACG AGA CCG AGT CGC TCA AG<br>CAT ATT TTT CCA GGG CTT CCT C<br>CCC TGT AAT TGG AAT GAG TCC A<br>ATC TGA TCG TCT TCG AAC CTC C
MTA1-FQ	Fw	1177→1194	TGC GAG AGC TGT TAC ACC
MTA1-RQ	Rv	1244→1227	RCG ACA CTG CAT GTT AGG[3]
DDX5-F	Fw		CTT GTC CTT GAT GAA GCA GA
DDX5-R	Rv		AGT CGC ACT CCA CAT TAG
MTA1-FL-F	Fw	194→212	TGT CAG TCT GCT ATA AGG C
MTA1dE4-F	Fw	178→190,<br>242→246	GAC AAG CAY GCA AGG GAA[3]
MTA1 −421	Rv	421→405	GGT AGG ACT TGA GCG AC
MTA1 +40	Fw	40→62	TTC GAG AAC TCC TCC AGC AAC CC
MTA1 -477	Rv	477→455	GGT CTT CTG CTG TGG GTC GTA GA
N-myc2 +780	Fw		GTT GAC GAG GAG GAA GAT GA
N-myc2 −948	Rv		GCA TAG TTG TGC TGC TGG TA

Fw: forward primer; Rv: reverse primer; EcoRI and XbaI endonuclease digestion sites are shown in bold. Start and stop codons are indicated by underlined and double underlined characters, respectively.

1: The primer was obtained from the FirstChoice RLM-RACE kit.

2: The position of the primers relative to our published *Marmota monax* MTA1 cDNA sequence (Accession number: JX891465.1).

3: Degenerate primer (symbol R = A or G nucleotide, symbol Y = C or T nucleotide).

Supplementary Table S2: Details of PCR amplification programs for molecular cloning

	Resulted region	Strategy	PCR program			
3′ RLM -RACE	1320 ↓ 2629	RACE-PCR	Step	Temperature (°C)	Time	No. of Cycle
			Initial Denature	95	3 min	1
			Denature Annealing Extension	95<br>57<br>72	30 sec<br>30 sec<br>30 sec	35
			Final extension	72	7 min	1
5′ RLM -RACE	1072 ↓ 1727	Slowdown PCR +	Step	Temperature (°C)	Time	No. of Cycle
			Initial Denature	98	3 min	1
			Denature Annealing Extension	98<br>66→52#<br>68	30 sec<br>30 sec<br>30 sec	45
			Denature Annealing Extension	98<br>57<br>68	30 sec<br>30 sec<br>30 sec	15*
			Final extension	68	7 min	1
wk-MTA1	1 ↓ 1244	Slowdown PCR +	As same as 5′ RLM-RACE			

All cycles were run in a generally reduced ramp rate at 2.5°C and especially a small cooling rate for reaching annealing temperature at 1.5°C.

#The annealing temperature is gradually decreased during the cycling process.

+A well-established PCR method, which allows the successful PCR-amplification of extremely GC-rich (> 83%) DNA targets.

*Additional cycles.
